# Radiomics-Based Image Phenotyping of Kidney Apparent Diffusion Coefficient Maps: Preliminary Feasibility & Efficacy

**DOI:** 10.3390/jcm11071972

**Published:** 2022-04-01

**Authors:** Lu-Ping Li, Alexander S. Leidner, Emily Wilt, Artem Mikheev, Henry Rusinek, Stuart M. Sprague, Orly F. Kohn, Anand Srivastava, Pottumarthi V. Prasad

**Affiliations:** 1 Department of Radiology, North Shore University HealthSystem, Evanston, IL 60201, USA; emilywilt7@gmail.com (E.W.); pprasad@northshore.org (P.V.P.); 2 Division of Nephrology and Hypertension, Center for Translational Metabolism and Health, Institute for Public Health and Medicine, Northwestern University Feinberg School of Medicine, Chicago, IL 60611, USA; alexander.leidner@northwestern.edu (A.S.L.); anand.srivastava@northwestern.edu (A.S.); 3 Center for Biomedical Imaging, New York University Langone Health, New York, NY 10016, USA; artemmikheev@gmail.com (A.M.); hr18@nyu.edu (H.R.); 4 Division of Nephrology, Department of Medicine, North Shore University HealthSystem, Evanston, IL 60201, USA; ssprague@northshore.org; 5 Division of Nephrology, Department of Medicine, Pritzker School of Medicine, University of Chicago, Chicago, IL 60637, USA; okohn@medicine.bsd.uchicago.edu

**Keywords:** kidney, MRI, radiomic, diffusion-weighted imaging, CKD, ADC

## Abstract

Given the central role of interstitial fibrosis in disease progression in chronic kidney disease (CKD), a role for diffusion-weighted MRI has been pursued. We evaluated the feasibility and preliminary efficacy of using radiomic features to phenotype apparent diffusion coefficient (ADC) maps and hence to the clinical classification(s) of the participants. The study involved 40 individuals (10 healthy and 30 with CKD (eGFR < 60 mL/min/1.73 m^2^)). Machine learning methods, such as hierarchical clustering and logistic regression, were used. Clustering resulted in the identification of two clusters, one including all individuals with CKD (*n* = 17), while the second one included all the healthy volunteers (*n* = 10) and the remaining individuals with CKD (*n* = 13), resulting in 100% specificity. Logistic regression identified five radiomic features to classify participants as with CKD vs. healthy volunteers, with a sensitivity and specificity of 93% and 70%, respectively, and an AUC of 0.95. Similarly, four radiomic features were able to classify participants as rapid vs. non-rapid CKD progressors among the 30 individuals with CKD, with a sensitivity and specificity of 71% and 43%, respectively, and an AUC of 0.75. These promising preliminary data should support future studies with larger numbers of participants with varied disease severity and etiologies to improve performance.

## 1. Introduction

Approximately 15% of adults in the United States have chronic kidney disease (CKD) [1], which increases the future risks of end-stage kidney disease, cardiovascular disease, and death. CKD is a heterogeneous condition with a wide spectrum of underlying etiologies, pathologic and clinical manifestations, and variable rates of progression. Estimated glomerular filtration rate (eGFR) and proteinuria are the two primary clinical indicators used to define and stage CKD [2], but they do not provide specificity regarding underlying histopathologic lesions, which also have prognostic value [3]. While low eGFR and high-grade proteinuria portend a high risk of adverse clinical outcomes, these biomarkers are insufficient to discern which individual patient with mild CKD will progress [4]. As a result, there is significant interest to explore alternate biomarkers of kidney disease to improve risk estimation of CKD progression.

Kidney tubulointerstitial fibrosis is recognized as a hallmark of progressive CKD [3]. By assessing the displacement of water molecules in tissue, diffusion-weighted magnetic resonance imaging (MRI) may detect kidney fibrosis [5]. Quantitative apparent diffusion coefficient (ADC) mapping, as assessed by diffusion-weighted MRI, has shown promise to quantify the magnitude of kidney cortical fibrosis. Lower levels of cortical ADC on diffusion-weighted MRI may indicate greater fibrosis [5]. In a prior study of individuals with diabetes and mild to moderate CKD (eGFR 51.2 ± 12.6 mL/min/1.73 m^2^), we found lower levels of ADC in individuals with CKD compared to age-matched healthy controls, but the difference did not reach statistical significance [6]. While there are significant data on the feasibility to incorporate multiple MRI measurements in clinical studies [6,7,8,9,10], these studies mainly focused on the mean values of the MRI biomarkers within regions of interest (ROI). Since imaging inherently captures spatial information, there is the potential for using a vast number of quantitative measures by processing at the level of an individual 3-dimensional sample known as a voxel. The use of radiomics, a method that extracts a large number of features from medical images using data-characterization algorithms, may uncover patterns, texture, or characteristics that may serve as digital fingerprints of disease [11]. Such methods have shown promise in individuals with liver disease [12], kidney cancer [13], and kidney transplants [14]. Based on our recent preliminary experience with selected radiomic features [15], we now have extended the analysis to include many more radiomic features in order to provide image phenotypes of the ADC maps and to verify whether the phenotypes correspond to clinical classification(s). We used machine learning techniques to highlight these latent features in radiomic data.

## 2. Materials and Methods

### 2.1. Study Population

We performed kidney diffusion-weighted MRI on individuals with and without CKD, as previously described [8]. We included adults ≥18 years old with diabetes (type 1 or 2) who had the ability and willingness to cooperate with the study protocol and did not have any contraindications for MRI study (claustrophobia, pacemakers, intra-cranial clips, or intraocular debris). CKD was defined by eGFR < 60 mL/min/1.73 m^2^. Healthy volunteers took no prescribed medications and had no history of CKD, diabetes mellitus, hypertension, coronary artery disease, congestive heart failure, liver disease, or autoimmune disease. Exclusion criteria were (1) significant co-morbid conditions that led the investigator to conclude that life expectancy was less than 1 year; (2) expected to progress to end-stage kidney disease, requiring the initiation of dialysis or a kidney transplant in the subsequent 24 months; (3) pregnant or nursing; (4) involved in any other interventional research protocol; (5) decompensated heart failure; (6) previous diagnosis of renal artery stenosis or ureteral obstruction; (7) CKD of other etiologies, such as glomerular disease, interstitial disease, and polycystic kidney disease; (8) chronic use of non-steroidal anti-inflammatory agents (NSAIDs); and (9) patients treated for anemia with ferumoxytol. Participants were recruited from NorthShore University HealthSystem and the University of Chicago. All participants provided written informed consent. The study protocol was approved by the Institutional Review Boards at NorthShore University HealthSystem and the University of Chicago and is in accordance with the principles of the Declaration of Helsinki. We enrolled 41 individuals with CKD and 13 healthy volunteers. We excluded 14 individuals for image analysis due to the presence of cysts (*n* = 10 CKD and *n* = 2 healthy volunteers) and 2 who were missing MRI data (*n* = 1 CKD and *n* = 1 healthy volunteer), which yielded a total sample size of 40 for this study (*n* = 30 CKD and *n* = 10 healthy).

### 2.2. MRI Acquisition

All MRI procedures were performed on a 3 Tesla whole-body scanner (Siemens Healthcare, Erlangen, Germany) using body array coils after an overnight fast. Participants were instructed to hold NSAIDs for 3 days prior, angiotensin-converting enzyme inhibitors/angiotensin II receptor blockers for 1 day prior, and loop diuretics on the day of the MRI. Medications were restarted following MRI data acquisition. Participants were scanned in a feet-first supine position. A spin-echo echo-planar imaging (EPI) technique with 3 directions of diffusion-sensitizing gradients were used to acquire diffusion-weighted images in five coronal planes during free breathing [6]. Diffusion-weighted MRI acquisition parameters used in this study were TE = 78 ms; FOV = 380–400 mm; TR = 3000 ms; bandwidth = 1628 Hz/pixel; matrix = 192 × 154; slice thickness = 5 mm; and b values = 200, 300, 500, 700, and 1000 s/mm^2^. Acquisitions were repeated 5 times and averaged to improve signal-to-noise ratio and to minimize motion artifact.

### 2.3. MRI Analysis

**Figure 1:**
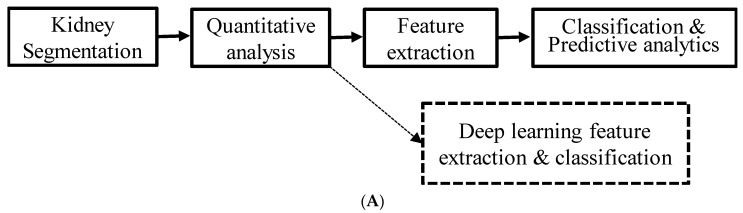

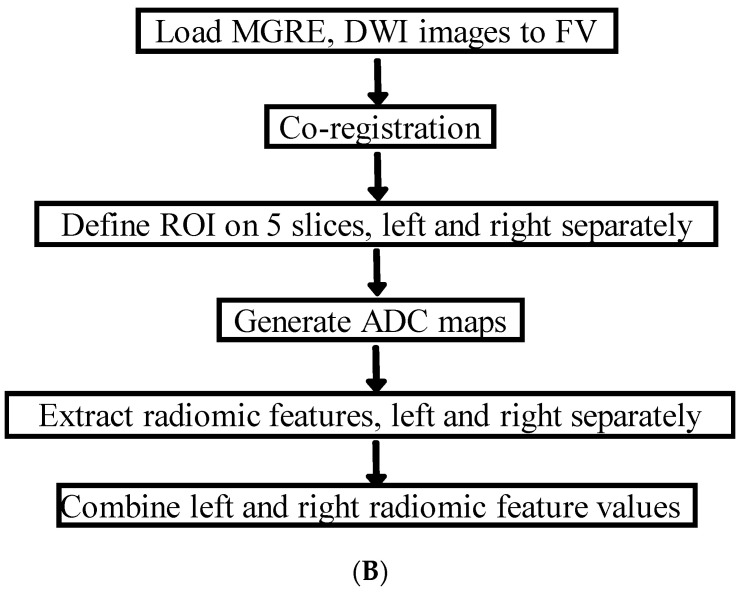
(**A**) High-level descriptions of the steps involved in the image analysis pipeline. The dotted lines indicate possible future extension. (**B**) A flow chart of kidney segmentation, quantitative analysis, and feature extraction using FireVoxel.

**Figure 2:**
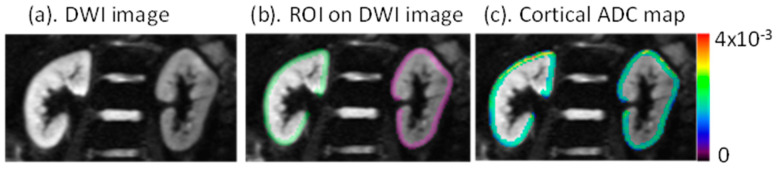
(**a**) Representative diffusion-weighted image. (**b**) Manually defined regions of interest (ROI) on left and right kidneys. Color identifies ROIs individually on the left and right kidneys. (**c**) ADC maps within the cortical ROI with color bar generated by FV, indicating relative ADC values in units of mm^2^/s.

The image analysis workflow is shown in Figure 1. Figure 1A shows the high-level description of the processing steps undertaken in the study. Figure 1B shows the specific steps involved in the image analysis using FireVoxel (FV), including the manual definition of regions of interest (ROI), quantitative parametric mapping, and radiomic feature extraction. Diffusion-weighted images were directly loaded to FV, and the images with different b values were co-registered by rigid transform to correct for respiratory motion. ROIs were defined on 5 slices of the left and right kidney cortices separately by author EW (Figure 2). Functional ROI maps were generated with FV on the left and right kidneys separately. A constant bin width of 4 × 10^−5^ for a range of ADC values from 0 to 4 × 10^−3^ mm^2^/s was used for all individual datasets. A total of 54 radiomics features were generated for left and right kidney ADC maps and averaged for a single representative value for each participant. The radiomic features from the right and left kidneys were highly correlated (Spearman *ρ* = 0.986 and *p* << 0.01), supporting their combination. FV software includes several categories of radiomic features. The radiomic features are separated into first-order (histogram features), such as central tendency parameters (mean, median, standard deviation, kurtosis, and skewness), gray level co-occurrence matrix (GLCM), and gray level run length matrix (GLRLM) textural features [16]. A recent publication [17] documented an agreement of texture features extracted from six software packages, including FireVoxel and 3D Slicer, with a radiomics extension based on the PyRadiomics library.

### 2.4. Exposures and Outcomes

In analyses that compared the 54 radiomic features in healthy volunteers and individuals with CKD, the exposure variable was CKD status (CKD vs. healthy volunteer). In analyses that investigated the associations of the 54 radiomic features with CKD status and CKD progressor status, the exposure variables were the radiomic features, and the outcomes were CKD status and rapid progressor status (defined as eGFR loss ≥ 3 mL/min/1.73 m^2^ per year), respectively.

### 2.5. Assessment of Clinical Information

At baseline, we collected participant demographics, diabetes mellitus status, body mass index (BMI), and eGFR. Blood pressure, proteinuria, and additional eGFR measurements were collected from the electronic medical record for CKD patients. We used the creatinine-based CKD Epidemiology Collaboration 2009 equation to calculate eGFR [18]. Proteinuria was quantified from a 24 h urine collection using the immunoturbidometric method.

### 2.6. Statistical Analysis

Descriptive statistics were summarized as count with percentages for categorical variables and mean ± standard deviation (SD) or median with interquartile range for normally distributed continuous variables or non-normally distributed continuous variables, respectively. To evaluate differences in radiomic parameters and clinical variables by CKD status, we used a *t*-test for normally distributed continuous variables and a Wilcoxon rank-sum test for non-normally distributed continuous variables. We used Spearman correlation coefficients to determine associations between non-normally distributed continuous variables.

We performed a hierarchical cluster analysis to identify distinct clusters of radiomic features using the Seaborn Clustermap module in Python 3.7 [19]. We transformed radiomic feature data using a Z-score prior to clustering according to the following: $Z=\frac{\left(x-mean\right)}{SD}$ [19]. Clinical characteristics were blinded for this computation. The sample distance between samples *u* and *v* was calculated using distance correlation: $1-\frac{\left(u-\bar{u}\right)\cdot \left(v-\bar{v}\right)}{{\left\|(u-\bar{u})\right\|}_{2\;}{\left\|(v-\bar{v})\right\|}_{2\;}}$ [20]. We created a Gaussian mixture model (GMM) with 1–10 clusters using a full variance matrix and plotted the Bayesian information criterion (BIC) versus the number of clusters to estimate the optimal number of clusters [21]. We utilized a hierarchical cluster map and dendrogram to determine cluster membership, qualitative inspection of trends in radiomic features, and to evaluate clinical features of radiomic clusters. To evaluate differences in radiomic parameters and clinical variables by cluster group, we performed a *t*-test for normally distributed continuous variables and a Wilcoxon rank-sum test for non-normally distributed continuous variables.

We also performed logistic regression to identify the most prominent features that distinguish those individuals with known CKD from the controls [20]. The regression model utilized forward selection of features, using the area under the curve of a receiver operating curve (AUC-ROC) to determine the best model. Terms were added sequentially to improve the AUC-ROC until there was no further improvement in the AUC-ROC. We reported the sensitivity, specificity, AUC-ROC, and accuracy of the regression models. All statistical tests were two-sided, and *p*-values < 0.05 were considered significant. In additional analyses, in individuals with CKD, we created 3 models using radiomics, clinical features, and a combination of both, to predict rapid versus non-rapid progressors.

## 3. Results

### 3.1. Study Participants

Baseline characteristics of the study cohort are shown in Table 1. The mean age of the individuals with CKD was slightly higher than that of the healthy volunteers (65.3 ± 9.6 vs. 58.1 ± 9.4 years; *p* = 0.05). The mean eGFR was lower in the individuals with CKD compared to the healthy volunteers (51.5 ± 12.2 vs. 88.6 ± 12.6) mL/min/1.73 m^2^; *p* < 0.001). The median urine protein excretion was 0.16 (IQR 0.02–0.25) g/day in individuals with CKD. The individuals with CKD had a higher BMI than the healthy volunteers (32.4 ± 7.5 vs. 25.8 ± 2.7 kg/m^2^; *p* = 0.01). During a mean follow-up time of 4.4 years, the mean annual eGFR slope was −0.5 ± 3.7 mL/min/1.73 m^2^ per year in the individuals with CKD. Seven participants (23%) experienced rapid CKD progression, defined as a mean annual loss of eGFR > 3 mL/min/1.73 m^2^ per year.

### 3.2. Correlations between Radiomic Features

**Figure 3:**
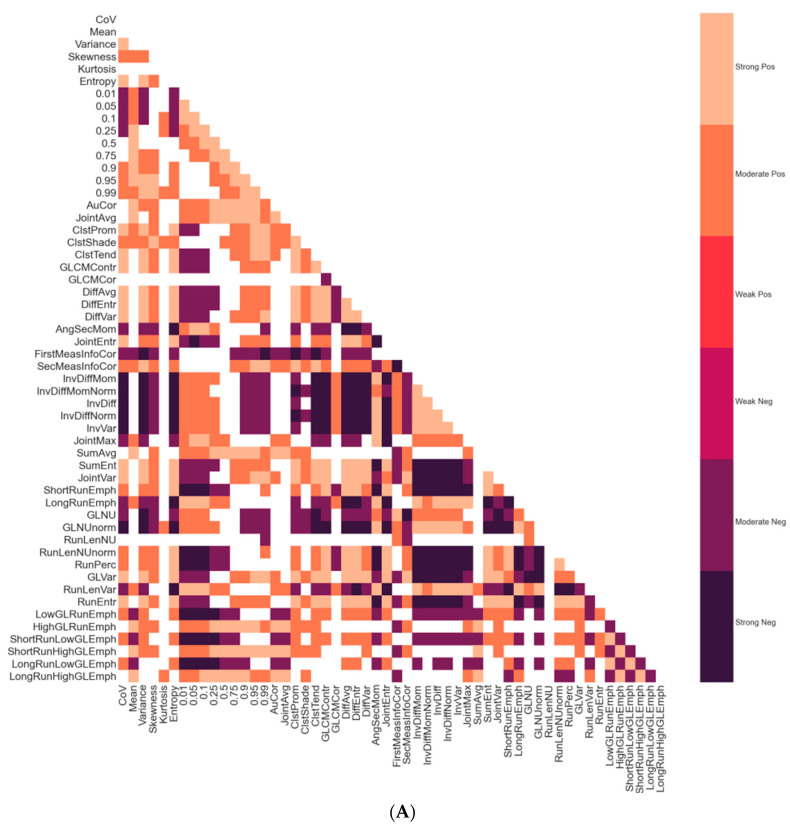

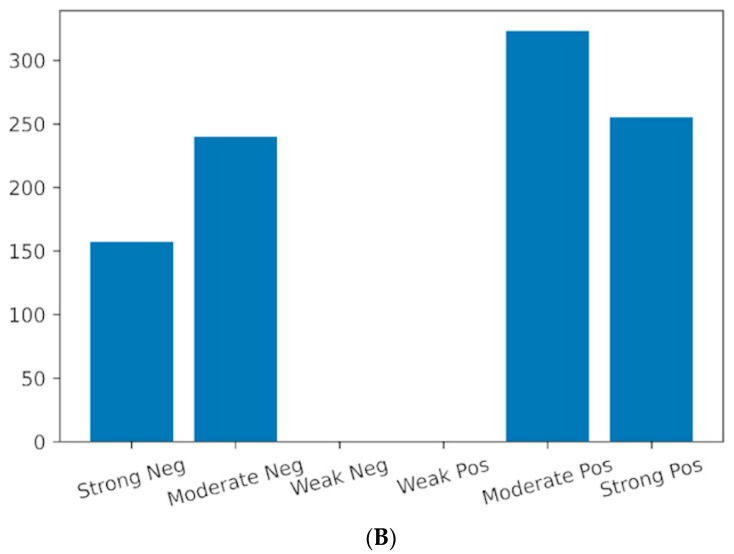
(**A**) Correlation map of radiomic features. Shown is a color-coded correlation map between all the radiomic features. Only statistically significant (*p* < 0.05) correlations are displayed. The color coding was based on the Spearman ± *ρ* values: strong (0.7 to 1.0), moderate (0.3 to 0.7), weak (0 to 0.3), and indicated as positive (pos) or negative (neg). (**B**) Radiomic feature correlation histogram. Histogram demonstrating the counts of each type of significant correlation.

Figure 3A is a correlation map between all 54 radiomic features. There was a high degree of statistically significant correlation between many features. Some features had strong negative correlations with one another, such as DiffAvg and InvDiff, and Variance and InvVar. Some first-order, GLCM, and GLRLM features shared derivations and had strong positive correlations with one another, such as such as Variance, GLVar, and DiffVar. There were 975 statistically significant correlations out of a potential 1431 between the 54 radiomic features, including 412 strong, 563 moderate, and no weak correlations (Figure 3B).

### 3.3. Radiomic Features in Individuals with CKD vs. Healthy Participants

Table 2 summarizes all 54 radiomic features by CKD status (15 first-order, 23 GLCM, and 16 GLRLM features). A total of 37 radiomic features differed by CKD status, including 8 first-order, 16 GLCM, and 13 GLRLM features. Three first-order features (CoV, 0.01, and 0.05), five GLCM (DiffAvg, InvDiffMom, InvDiff, InvDiffNorm, and InvVar), and one GLRLM feature (GLNU) had strong statistically significant correlations (*p* < 0.001).

### 3.4. Correlations between Radiomic Features and Clinical Parameters

**Figure 4:**
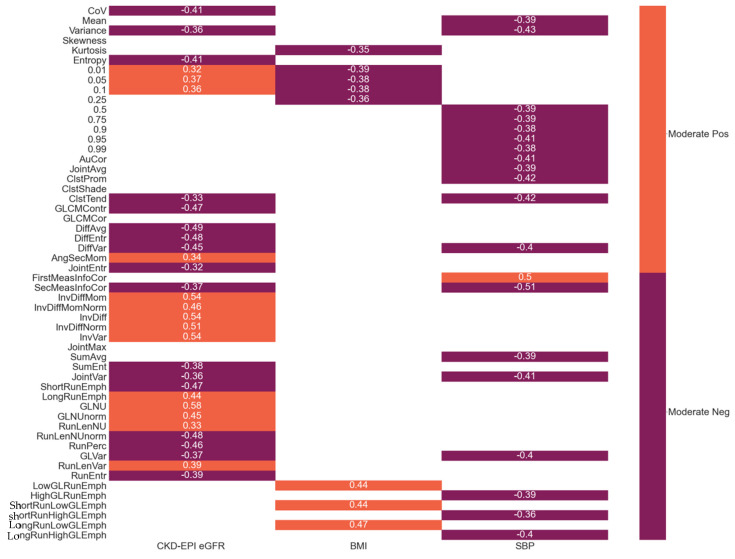
Correlation map of radiomic and clinical features. Shown is a color-coded correlation map between radiomic and clinical features. Only statistically significant (*p* < 0.05) correlations are displayed. There were no significant correlations in the categories of age, sex, diastolic blood pressure, eGFR slope, 24 h urine protein, or blood glucose. Spearman *ρ* value of correlations is displayed. The color coding was based on the Spearman ± *ρ* values; however, all correlations were moderate (0.3 to 0.7) and indicated as positive (pos) or negative (neg). Abbreviations: CKD: chronic kidney disease; CKD-EPI: CKD Epidemiology Collaboration; eGFR: estimated glomerular filtration rate; SBP: systolic blood pressure; BMI: body mass index.

Figure 4 is a correlation map between the radiomic features and clinical parameters of the study participants. Of all radiomic features, there were 31, 20, and 8 statistically significant correlations with eGFR, systolic blood pressure (SBP), and BMI, respectively. Among associations between radiomic features and eGFR, there were 6, 15, and 10 first-order, GLCM, and GLRLM features, respectively, that were statistically significant. GLNU had the strongest correlation with eGFR (*ρ* = 0.58 and *p* < 0.001). LongRunLowGLEmph had the strongest correlation with BMI (*ρ* = 0.47 and *p* = 0.002). SecMeasInfoCor had the strongest correlation with SBP (*ρ* = −0.51 and *p* = 0.004). 

### 3.5. Hierarchical Clustering by Radiomic Features

**Figure 5:**
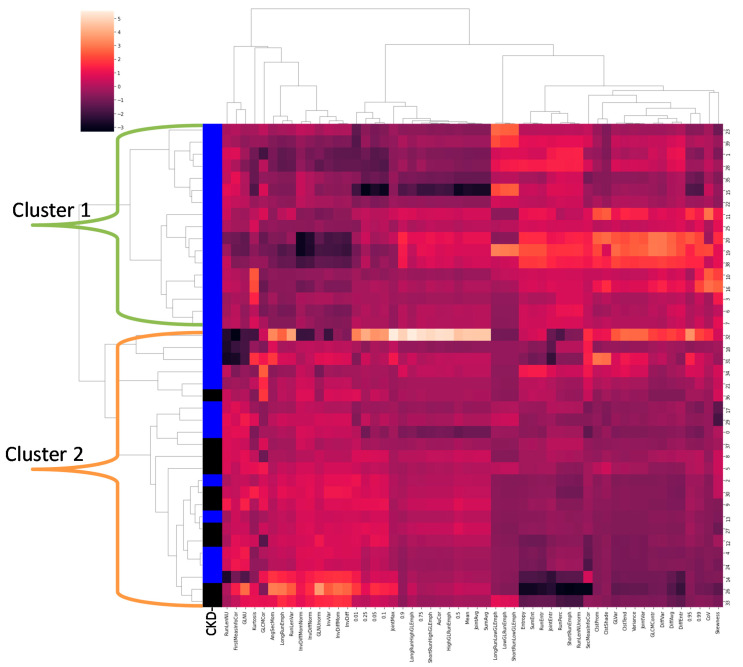
Hierarchical clustering of radiomic features. Shown are Z-score normalized values of each individual radiomic feature in each column, with each participant as a unique row. The first column represents the presence (blue) or absence (black) of CKD in the subject. The clustering shows two distinct phenotypes. Phenotype 1 (Cluster 1) can be identified as CKD, while phenotype 2 (Cluster 2) includes both CKD and controls, possibly indicating early changes. Cluster 1 had more negative Z-scores (darker red) in many of the first 29 feature columns and more positive Z-scores (lighter red) in the following 25 feature columns.

**Figure 6:**
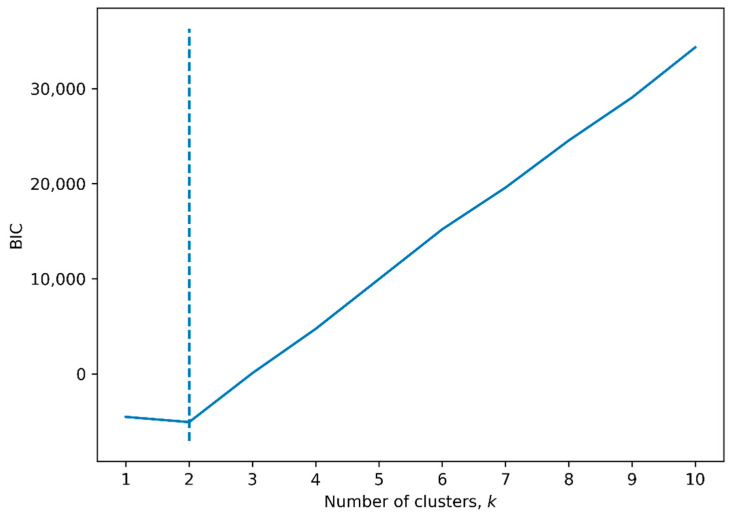
BIC for number of clusters in a GMM. Shown is the Bayesian information criterion (BIC) versus the number of clusters in a Gaussian mixture model (GMM). The dotted line indicates the minimum BIC at two clusters.

Figure 5 demonstrates the results of hierarchical clustering in a dendrogram and heat map. Figure 6 demonstrates the BIC score of consecutive clusters, showing a worse model fit (higher score) for GMM models using more than two clusters. The figure demonstrates a clinical delineation (blue/black column representing CKD) between the two most dissimilar clusters. Cluster 1 (the upper half of the dendrogram) consisted of 17 individuals, all with CKD (100%), and cluster 2 (the lower half of the dendrogram) included 23 individuals, 13 of which had CKD (57%) (*p* = 0.001).

Table 3 shows the radiomic and clinical parameters stratified by cluster. The mean eGFR of cluster 1 and cluster 2 was 49.8 ± 11.5 and 68.9 ± 21.7 mL/min/1.73 m^2^, respectively (*p* = 0.002). There were no significant differences in eGFR slope, BMI, SBP, DBP, age, or blood glucose by cluster. There were 39 radiomic parameters that differed between the clusters. There were three measurements that were significantly different between the CKD and healthy groups that were not different between clusters, including 0.5, FirstMeasInfoCor, and LongRunHighGLEmph. There were five measurements that were significantly different between clusters that were not different between the CKD and healthy groups, including skewness, 0.99, JointMax, GLCMCor, and ClstProm.

### 3.6. Radiomics-Based Prediction of CKD and CKD Progression

To predict CKD vs. healthy volunteers in the entire cohort (*n* = 40), the stepwise inclusion of the following features improved the AUC-ROC: GLNU, ShortRunHighGLEmph, GLCMContr, kurtosis, and skewness. For predicting CKD, the model’s sensitivity was 93%, specificity was 70%, and the AUC-ROC was 0.95.

The rapid and non-rapid progressors had a mean annual eGFR slope of −5.27 ± 2.61 and 0.92 ± 2.57 mL/min/1.73 m^2^ per year, respectively (*p* < 0.001). To predict rapid progressor vs. non-rapid progressor status among individuals with CKD (*n* = 30), we created three models using radiomics, baseline clinical features, and both radiomics and baseline clinical features (Table 4). In the radiomics model, the stepwise inclusion of the following features improved the AUC-ROC: GLCMContr, SumEnt, CoV, and FirstMeasInfoCor. For predicting rapid progressors, this model’s sensitivity was 71%, specificity was 43%, and the AUC-ROC was 0.75. In the baseline clinical features model, the stepwise inclusion of the following features improved the AUC-ROC: 24 h urine protein excretion and sex. For predicting rapid progressors, this model’s sensitivity was 57%, specificity was 91%, and the AUC-ROC was 0.94. In the combination model, the stepwise inclusion of the following features improved the AUC-ROC: 24 h urine protein excretion, sex, and AuCor. For predicting rapid progressors, this model’s sensitivity was 57%, specificity was 96%, and the AUC-ROC was 0.96.

## 4. Discussion

In this preliminary feasibility study, we identified significant differences in radiomic features derived from kidney ADC maps in individuals with mild to moderate CKD and healthy volunteers. Using unsupervised hierarchical clustering, a machine learning approach, we were able to identify two clusters with distinct radiomic signatures and clinical phenotypes—one cluster included individuals with CKD and another cluster that had a mixture of individuals with CKD and healthy volunteers. Finally, a logistic regression model was able to identify five radiomic features that distinguished individuals with CKD from healthy volunteers. Our modeling approach that included four radiomic features had a limited ability to classify individuals who experienced rapid CKD progression from individuals without rapid CKD progression. Proteinuria was the strongest clinical variable associated with rapidly progressive CKD, which was previously reported [22]. In this exploratory study, radiomic features were able to slightly increase discrimination of rapid progressors from non-rapid progressors beyond clinical variables. This may indicate a need for larger studies to evaluate their ability to improve upon already existing prediction models. Taken together, our findings should stimulate further research to determine whether radiomic features are able to better phenotype individuals with CKD and add prognostic value independent of clinical characteristics.

Kidney cortical fibrosis is recognized as a hallmark of progressive CKD [3,23]. Quantitative ADC mapping, as assessed by diffusion-weighted MRI, demonstrated promise to non-invasively identify kidney cortical fibrosis [5]. However, reliance on a single imaging biomarker may miss opportunities to utilize more of the imaging data that capture additional phenotypic signatures of disease. While radiomic-based phenotyping approaches have been used in multiple diseased organ systems, there have been relatively few performed in CKD [11,14]. Our results are the first, to our knowledge, to demonstrate the feasibility of radiomics-based analysis applied to quantitative kidney diffusion-weighted MRI. In a prior report, we showed that individuals with mild to moderate CKD had lower ADC values, suggestive of increased fibrosis, compared to age-matched healthy volunteers, but these findings did not reach statistical significance [6]. In this study, we were able to identify a number of radiomic features that were significantly different between the same two groups. We further demonstrated that clustering by radiomic features, agnostic to clinical variables, was able to separate the participants into a homogenous cluster of individuals with CKD and a more heterogeneous cluster comprised of healthy volunteers and individuals with CKD. One potential reason for why one cluster consisted of healthy volunteers and individuals with CKD, indicative of similar vectors of radiomic features, is that these individuals may share a similar clinical phenotype that may not be captured by the single clinical variable (eGFR) used to classify participants as with CKD or healthy. If confirmed to be true, the implication of this finding may suggest that these individuals with CKD have a lower likelihood of CKD progression, since they are more similar to healthy volunteers, or that some of the healthy volunteers may be at risk for CKD. Future research is warranted to determine if radiomic features can identify clinically useful sub-phenotypes of individuals with CKD.

Prior studies have demonstrated promise for the use of radiomics to identify signatures of disease independent of clinical variables [24]. A recent analysis in individuals with non-alcoholic fatty liver disease demonstrated that texture analysis, a form of radiomics that captures spatial heterogeneity of tissue, outperformed clinical variables to identify individuals with advanced liver fibrosis [14]. Texture analysis of computed tomography (CT) scans predicted progression of renal cell carcinoma [25], and radiomic analysis of MRI for breast cancer predicted tumor receptor status [26]. Similarly, radiomic measurements of kidney MRIs may serve as a prognostic marker in a heterogeneous disease like CKD. Our logistic regression model that consisted of five radiomic features was able to differentiate individuals with CKD from healthy volunteers with excellent discrimination. Similarly, four radiomic features were able to moderately discriminate individuals who experienced rapid CKD progression from individuals who did not experience rapid CKD progression. While it is known that proteinuria is a strong predictor of CKD progression at the population level [22], it may not be able to identify which individual patient will experience rapid progression. In this study, radiomic features alone did not outperform clinical features, but AuCor was able to provide a mild increase in AUC-ROC in combination with clinical variables. These findings underscore the need to further test whether radiomic features are able to improve risk prediction of rapid CKD progression in larger follow-up studies. While some of the features correlated with one another, the stepwise addition of these radiomic features improved the ability of each respective model to predict CKD and rapid progression status. Larger studies with a representative spectrum of disease severity, a variety of CKD etiologies, and robust longitudinal phenotyping, are required to further identify disease sub-clusters and to identify individuals at high risk of CKD progression independent of clinical variables.

The strengths of our study include the use of individuals with CKD and age-matched healthy volunteers and the use of advanced image processing tools to generate radiomic features from kidney ADC maps. We also used kidney ADC parametric maps that have less inter-study variability than signal intensity, which can be influenced by instrument and acquisition parameters specific to each collection. Our study has several limitations that warrant consideration, as well. Our preliminary feasibility study utilized existing data in a small number of individuals, and future studies that include more individuals across the spectrum of CKD severity, with longer follow-ups, are needed to fully evaluate the efficacy of these approaches by allowing for appropriate size for cross validation, external validation, and comprehensive multivariable adjustment of potential confounders. We only used the 54 radiomic features available in FV, while many other encoding methods exist [11], which will require investigation in follow-up studies. A lack of reproducibility data for radiomic features and a need for validation of quantitative imaging studies is a limitation. Future investigators must exercise caution and become fully familiar with radiomics workflow processing details [27]. We measured eGFR in our healthy volunteers, but some of these individuals had eGFR values that may be considered early or mild CKD, which we did not confirm with proteinuria measurements. We only included manually defined cortical ADC ROIs, and future studies should consider deep learning methods to segment renal parenchyma [28] for feature extraction and classification. Since the participants did not undergo a native kidney biopsy, we were unable to assess whether the radiomic features provide better estimates of underlying kidney cortical fibrosis than the single mean ADC values, which will require further study in initiatives such as the Kidney Precision Medicine Project.

## 5. Conclusions

In conclusion, a number of radiomic features showed significant differences between individuals with CKD and healthy volunteers on kidney ADC maps, even though mean ADC values were not significantly different. Importantly, our preliminary data support the use of machine learning-based clustering techniques of radiomic features of quantitative MRI parametric maps to provide additional phenotyping of individuals with CKD. Our findings suggest the need for larger prospective studies that incorporate radiomics-based approaches to analyze kidney MRI as a tool to identity individuals at high risk of rapid CKD progression, in an effort to improve upon existing prediction models.

## Figures and Tables

**Table 1:** Baseline characteristics of healthy and CKD groups.

	Healthy (*n* = 10)	CKD (*n* = 30)	*p*-Value
Female or Male?	0.4	0.5	0.86
Age (years)	58.1 ± 9.4	65.3 ± 9.6	0.05
SBP (mmHg)		133.87 ± 15.9	
DBP (mmHg)		67.9 ± 10.6	
CKD-EPI eGFR (mL/min/1.73 m^2^)	88.6 ± 12.6	51.5 ± 12.2	<0.001
BMI (kg/m^2^)	25.8 ± 2.7	32.4 ± 7.5	0.01
eGFR slope (mL/min/1.73 m^2^/year)		−0.53 ± 3.68	
24 h urine protein excretion (gm)		0.16 (0.018–0.253)	
Blood glucose (mg/dL)		149.6 ± 68.0	

Note: Mean values with standard deviation (SD) are reported for normally distributed variables; otherwise, median values with interquartile range (IQR) are reported: CKD: chronic kidney disease; CKD-EPI: the CKD Epidemiology Collaboration; eGFR: estimated glomerular filtration rate; SBP: systolic blood pressure; DBP: diastolic blood pressure; BMI: body mass index.

**Table 2:** Differences in radiomic features between two groups defined by eGFR.

	Healthy (*n* = 10)	CKD (*n* = 30)	*p*-Value	
CoV	1.64 × 10^−1^ (1.58 × 10^−1^–1.94 × 10^−1^)	2.41 × 10^−1^ (2.01 × 10^−1^–2.97 × 10^−1^)	0.001	1st order
Mean	1.83 × 10^−3^ (1.77 × 10^−3^–1.87 × 10^−3^)	1.75 × 10^−3^ (1.65 × 10^−3^–1.82 × 10^−3^)	0.092	
Variance	9.77 × 10^−8^ (8.09 × 10^−8^–1.19 × 10^−7^)	1.68 × 10^−7^ (1.12 × 10^−7^–2.29 × 10^−7^)	0.006	
Skewness	−1.22 × 10^−1^ (−5.36 × 10^−1^–2.70 × 10^−1^)	−3.02 × 10^−2^ (−4.68 × 10^−1^–5.80 × 10^−1^)	0.288	
Kurtosis	2.89 × 10^0^ (1.77 × 10^0^–3.77 × 10^0^)	1.65 × 10^0^ (1.23 × 10^0^–2.70 × 10^0^)	0.126	
Entropy	3.38 × 10^0^ (3.27 × 10^0^–3.46 × 10^0^)	3.59 × 10^0^ (3.39 × 10^0^–3.69 × 10^0^)	0.006	
0.01	9.75 × 10^−4^ (9.12 × 10^−4^–9.92 × 10^−4^)	7.11 × 10^−4^ (5.77 × 10^−4^–8.09 × 10^−4^)	0.000	
0.05	1.32 × 10^−3^ (1.25 × 10^−3^–1.37 × 10^−3^)	1.10 × 10^−3^ (9.55 × 10^−4^–1.15 × 10^−3^)	0.000	
0.1	1.47 × 10^−3^ (1.38 × 10^−3^–1.52 × 10^−3^)	1.30 × 10^−3^ (1.17 × 10^−3^–1.36 × 10^−3^)	0.001	
0.25	1.68 × 10^−3^ (1.60 × 10^−3^–1.74 × 10^−3^)	1.54 × 10^−3^ (1.45 × 10^−3^–1.61 × 10^−3^)	0.007	
0.5	1.83 × 10^−3^ (1.76 × 10^−3^–1.87 × 10^−3^)	1.75 × 10^−3^ (1.67 × 10^−3^–1.82 × 10^−3^)	0.027	
0.75	1.97 × 10^−3^ (1.95 × 10^−3^–2.03 × 10^−3^)	1.94 × 10^−3^ (1.88 × 10^−3^–2.01 × 10^−3^)	0.274	
0.9	2.16 × 10^−3^ (2.08 × 10^−3^–2.23 × 10^−3^)	2.13 × 10^−3^ (2.03 × 10^−3^–2.26 × 10^−3^)	1.000	
0.95	2.32 × 10^−3^ (2.20 × 10^−3^–2.37 × 10^−3^)	2.30 × 10^−3^ (2.14 × 10^−3^–2.67 × 10^−3^)	0.685	
0.99	2.59 × 10^−3^ (2.50 × 10^−3^–2.70 × 10^−3^)	2.80 × 10^−3^ (2.41 × 10^−3^–3.30 × 10^−3^)	0.235	
AuCor	2.17 × 10^3^ (2.08 × 10^3^–2.31 × 10^3^)	2.02 × 10^3^ (1.84 × 10^3^–2.21 × 10^3^)	0.179	gray level co-occurrence matrix
JointAvg	4.63 × 10^1^ (4.49 × 10^1^–4.76 × 10^1^)	4.44 × 10^1^ (4.22 × 10^1^–4.64 × 10^1^)	0.098	
ClstProm	2.29 × 10^5^ (1.58 × 10^5^–2.70 × 10^5^)	5.98 × 10^5^ (1.95 × 10^5^–1.09 × 10^6^)	0.053	
ClstShade	−1.23 × 10^2^ (−5.89 × 10^2^–1.55 × 10^3^)	9.08 × 10^2^ (−8.14 × 10^2^–7.55 × 10^3^)	0.492	
ClstTend	1.98 × 10^2^ (1.63 × 10^2^–2.31 × 10^2^)	3.12 × 10^2^ (2.10 × 10^2^–4.64 × 10^2^)	0.008	
GLCMContr	3.42 × 10^1^ (3.28 × 10^1^–4.07 × 10^1^)	6.65 × 10^1^ (4.95 × 10^1^–9.84 × 10^1^)	0.001	
GLCMCor	7.00 × 10^−1^ (6.08 × 10^−1^–7.14 × 10^−1^)	6.22 × 10^−1^ (5.92 × 10^−1^–6.75 × 10^−1^)	0.190	
DiffAvg	4.22 × 10^0^ (4.04 × 10^0^–4.46 × 10^0^)	5.49 × 10^0^ (4.93 × 10^0^–6.81 × 10^0^)	0.001	
DiffEntr	3.55 × 10^0^ (3.50 × 10^0^–3.65 × 10^0^)	3.91 × 10^0^ (3.72 × 10^0^–4.22 × 10^0^)	0.001	
DiffVar	1.66 × 10^1^ (1.52 × 10^1^–1.96 × 10^1^)	3.34 × 10^1^ (2.03 × 10^1^–4.76 × 10^1^)	0.002	
AngSecMom	3.57 × 10^−3^ (3.36 × 10^−3^–4.18 × 10^−3^)	2.59 × 10^−3^ (2.07 × 10^−3^–3.64 × 10^−3^)	0.021	
JointEntr	8.69 × 10^0^ (8.46 × 10^0^–8.89 × 10^0^)	9.13 × 10^0^ (8.63 × 10^0^–9.36 × 10^0^)	0.025	
FirstMeasInfoCor	−2.02 × 10^−1^ (−2.12 × 10^−1^–−1.93 × 10^−1^)	−2.27 × 10^−1^ (−2.70 × 10^−1^–−1.95 × 10^−1^)	0.042	
SecMeasInfoCor	9.24 × 10^−1^ (9.21 × 10^−1^–9.26 × 10^−1^)	9.49 × 10^−1^ (9.25 × 10^−1^–9.62 × 10^−1^)	0.018	
InvDiffMom	2.33 × 10^−1^ (2.30 × 10^−1^–2.50 × 10^−1^)	1.84 × 10^−1^ (1.66 × 10^−1^–2.18 × 10^−1^)	0.001	
InvDiffMomNorm	9.97 × 10^−1^ (9.96 × 10^−1^–9.97 × 10^−1^)	9.94 × 10^−1^ (9.91 × 10^−1^–9.95 × 10^−1^)	0.001	
InvDiff	3.23 × 10^−1^ (3.20 × 10^−1^–3.38 × 10^−1^)	2.73 × 10^−1^ (2.52 × 10^−1^–3.08 × 10^−1^)	0.000	
InvDiffNorm	9.61 × 10^−1^ (9.59 × 10^−1^–9.63 × 10^−1^)	9.50 × 10^−1^ (9.40 × 10^−1^–9.55 × 10^−1^)	0.001	
InvVar	2.40 × 10^−1^ (2.38 × 10^−1^–2.58 × 10^−1^)	1.90 × 10^−1^ (1.63 × 10^−1^–2.26 × 10^−1^)	0.001	
JointMax	1.16 × 10^−2^ (1.09 × 10^−2^–1.33 × 10^−2^)	9.03 × 10^−3^ (7.42 × 10^−3^–1.19 × 10^−2^)	0.065	
SumAvg	9.27 × 10^1^ (8.98 × 10^1^–9.53 × 10^1^)	8.87 × 10^1^ (8.44 × 10^1^–9.27 × 10^1^)	0.098	
SumEnt	5.69 × 10^0^ (5.49 × 10^0^–5.76 × 10^0^)	5.96 × 10^0^ (5.65 × 10^0^–6.09 × 10^0^)	0.016	
JointVar	5.79 × 10^1^ (4.79 × 10^1^–7.20 × 10^1^)	9.90 × 10^1^ (6.54 × 10^1^–1.38 × 10^2^)	0.007	
ShortRunEmph	9.49 × 10^−1^ (9.45 × 10^−1^–9.51 × 10^−1^)	9.62 × 10^−1^ (9.53 × 10^−1^–9.64 × 10^−1^)	0.002	gray level run length matrix
LongRunEmph	1.23 × 10^0^ (1.22 × 10^0^–1.25 × 10^0^)	1.17 × 10^0^ (1.15 × 10^0^–1.21 × 10^0^)	0.002	
GLNU	6.67 × 10^1^ (5.98 × 10^1^–6.86 × 10^1^)	4.43 × 10^1^ (3.61 × 10^1^–5.25 × 10^1^)	0.000	
GLNUnorm	4.26 × 10^−2^ (4.16 × 10^−2^–4.79 × 10^−2^)	3.51 × 10^−2^ (3.04 × 10^−2^–4.12 × 10^−2^)	0.007	
RunLenNU	1.30 × 10^3^ (1.17 × 10^3^–1.41 × 10^3^)	1.21 × 10^3^ (1.06 × 10^3^–1.32 × 10^3^)	0.142	
RunLenNUnorm	8.76 × 10^−1^ (8.67 × 10^−1^–8.80 × 10^−1^)	9.04 × 10^−1^ (8.83 × 10^−1^–9.10 × 10^−1^)	0.001	
RunPerc	9.33 × 10^−1^ (9.27 × 10^−1^–9.36 × 10^−1^)	9.49 × 10^−1^ (9.38 × 10^−1^–9.53 × 10^−1^)	0.002	
GLVar	6.32 × 10^1^ (5.21 × 10^1^–7.59 × 10^1^)	1.07 × 10^2^ (7.21 × 10^1^–1.44 × 10^2^)	0.005	
RunLenVar	8.05 × 10^−2^ (7.40 × 10^−2^–8.95 × 10^−2^)	6.20 × 10^−2^ (5.36 × 10^−2^–7.46 × 10^−2^)	0.006	
RunEntr	5.25 × 10^0^ (5.10 × 10^0^–5.33 × 10^0^)	5.45 × 10^0^ (5.23 × 10^0^–5.60 × 10^0^)	0.014	
LowGLRunEmph	5.54 × 10^−4^ (5.17 × 10^−4^–6.23 × 10^−4^)	7.54 × 10^−4^ (6.06 × 10^−4^–1.56 × 10^−3^)	0.015	
HighGLRunEmph	2.17 × 10^3^ (2.07 × 10^3^–2.30 × 10^3^)	2.02 × 10^3^ (1.83 × 10^3^–2.23 × 10^3^)	0.179	
ShortRunLowGLEmph	5.29 × 10^−4^ (4.96 × 10^−4^–6.00 × 10^−4^)	7.26 × 10^−4^ (5.82 × 10^−4^–1.50 × 10^−3^)	0.014	
ShortRunHighGLEmph	2.03 × 10^3^ (1.97 × 10^3^–2.18 × 10^3^)	1.93 × 10^3^ (1.75 × 10^3^–2.10 × 10^3^)	0.235	
LongRunLowGLEmph	6.65 × 10^−4^ (6.46 × 10^−4^–7.26 × 10^−4^)	8.87 × 10^−4^ (7.09 × 10^−4^–1.64 × 10^−3^)	0.025	
LongRunHighGLEmph	2.71 × 10^3^ (2.56 × 10^3^–2.89 × 10^3^)	2.47 × 10^3^ (2.18 × 10^3^–2.66 × 10^3^)	0.049	

Note: Median values with inter-quartile range (IQR) are reported. *p* < 0.05 is considered significant.

**Table 3:** Differences in clinical and radiomic features between two phenotypes.

	Cluster 1 (*n* = 17)	Cluster 2 (*n* = 23)	*p*-Value	
CKD	1.0 ± 0.0	0.6 ± 0.5	0.001	clinical
Female or Male?	0.4 ± 0.5	0.3 ± 0.5	0.689	
Age (years)	65.1 ± 10.3	62.3 ± 9.7	0.376	
SBP (mmHg)	135.4 ± 16.5	131.6 ± 15.5	0.527	
DBP (mmHg)	67.1 ± 12.6	69.1 ± 7.7	0.615	
CKD-EPI eGFR (mL/min/1.73 m^2^)	49.8 ± 11.5	68.9 ± 21.7	0.002	
BMI (kg/m^2^)	33.2 ± 8.1	29.0 ± 6.0	0.067	
eGFR slope (mL/min/1.73 m^2^/year)	−0.3 ± 4.4	−0.8 ± 2.6	0.745	
24 h urine protein excretion (gm)	0.1 ± 0.0	0.2 ± 0.0	0.194	
Blood glucose (mg/dL)	157.9 ± 74.4	138.8 ± 59.8	0.454	
CoV	2.63 × 10^−1^ (2.37 × 10^−1^–3.18 × 10^−1^)	1.86 × 10^−1^ (1.59 × 10^−1^–2.05 × 10^−1^)	0.000	1st order
Mean	1.72 × 10^−3^ (1.61 × 10^−3^–1.82 × 10^−3^)	1.79 × 10^−3^ (1.72 × 10^−3^–1.84 × 10^−3^)	0.245	
Variance	1.99 × 10^−7^ (1.67 × 10^−7^–2.49 × 10^−7^)	9.84 × 10^−8^ (8.25 × 10^−8^–1.23 × 10^−7^)	0.000	
Skewness	5.78 × 10^−1^ (−2.90 × 10^−1^–8.08 × 10^−1^)	−1.34 × 10^−1^ (−5.31 × 10^−1^–2.35 × 10^−1^)	0.014	
Kurtosis	1.93 × 10^0^ (1.24 × 10^0^–3.31 × 10^0^)	1.67 × 10^0^ (1.30 × 10^0^–3.26 × 10^0^)	0.989	
Entropy	3.66 × 10^0^ (3.60 × 10^0^–3.75 × 10^0^)	3.37 × 10^0^ (3.31 × 10^0^–3.45 × 10^0^)	0.000	
0.01	5.86 × 10^−4^ (4.48 × 10^−4^–7.17 × 10^−4^)	9.24 × 10^−4^ (7.82 × 10^−4^–9.87 × 10^−4^)	0.000	
0.05	9.77 × 10^−4^ (9.14 × 10^−4^–1.12 × 10^−3^)	1.25 × 10^−3^ (1.12 × 10^−3^–1.33 × 10^−3^)	0.000	
0.1	1.19 × 10^−3^ (1.14 × 10^−3^–1.31 × 10^−3^)	1.37 × 10^−3^ (1.32 × 10^−3^–1.47 × 10^−3^)	0.000	
0.25	1.52 × 10^−3^ (1.43 × 10^−3^–1.58 × 10^−3^)	1.61 × 10^−3^ (1.54 × 10^−3^–1.68 × 10^−3^)	0.003	
0.5	1.72 × 10^−3^ (1.65 × 10^−3^–1.80 × 10^−3^)	1.80 × 10^−3^ (1.74 × 10^−3^–1.84 × 10^−3^)	0.057	
0.75	1.94 × 10^−3^ (1.86 × 10^−3^–2.02 × 10^−3^)	1.96 × 10^−3^ (1.90 × 10^−3^–2.00 × 10^−3^)	0.613	
0.9	2.20 × 10^−3^ (2.06 × 10^−3^–2.33 × 10^−3^)	2.13 × 10^−3^ (2.04 × 10^−3^–2.21 × 10^−3^)	0.245	
0.95	2.40 × 10^−3^ (2.28 × 10^−3^–2.69 × 10^−3^)	2.26 × 10^−3^ (2.13 × 10^−3^–2.36 × 10^−3^)	0.109	
0.99	3.05 × 10^−3^ (2.65 × 10^−3^–3.56 × 10^−3^)	2.56 × 10^−3^ (2.42 × 10^−3^–2.81 × 10^−3^)	0.029	
AuCor	1.99 × 10^3^ (1.73 × 10^3^–2.22 × 10^3^)	2.14 × 10^3^ (1.95 × 10^3^–2.26 × 10^3^)	0.404	gray level co-occurrence matrix
JointAvg	4.40 × 10^1^ (4.10 × 10^1^–4.64 × 10^1^)	4.55 × 10^1^ (4.38 × 10^1^–4.68 × 10^1^)	0.256	
ClstProm	7.26 × 10^5^ (4.97 × 10^5^–1.77 × 10^6^)	2.05 × 10^5^ (1.14 × 10^5^–2.93 × 10^5^)	0.001	
ClstShade	5.03 × 10^3^ (−1.49 × 10^3^–9.33 × 10^3^)	−2.36 × 10^1^ (−7.35 × 10^2^–1.93 × 10^3^)	0.318	
ClstTend	3.76 × 10^2^ (3.05 × 10^2^–4.71 × 10^2^)	2.01 × 10^2^ (1.63 × 10^2^–2.46 × 10^2^)	0.000	
GLCMContr	9.19 × 10^1^ (7.35 × 10^1^–1.04 × 10^2^)	3.74 × 10^1^ (3.33 × 10^1^–5.00 × 10^1^)	0.000	
GLCMCor	6.06 × 10^−1^ (5.75 × 10^−1^–6.36 × 10^−1^)	6.80 × 10^−1^ (6.02 × 10^−1^–7.18 × 10^−1^)	0.009	
DiffAvg	6.74 × 10^0^ (5.86 × 10^0^–7.44 × 10^0^)	4.39 × 10^0^ (4.11 × 10^0^–4.99 × 10^0^)	0.000	
DiffEntr	4.20 × 10^0^ (3.99 × 10^0^–4.32 × 10^0^)	3.61 × 10^0^ (3.53 × 10^0^–3.74 × 10^0^)	0.000	
DiffVar	4.29 × 10^1^ (3.42 × 10^1^–5.68 × 10^1^)	1.77 × 10^1^ (1.52 × 10^1^–2.25 × 10^1^)	0.000	
AngSecMom	2.13 × 10^−3^ (1.90 × 10^−3^–2.33 × 10^−3^)	3.65 × 10^−3^ (3.23 × 10^−3^–4.52 × 10^−3^)	0.000	
JointEntr	9.35 × 10^0^ (9.22 × 10^0^–9.52 × 10^0^)	8.62 × 10^0^ (8.30 × 10^0^–8.85 × 10^0^)	0.000	
FirstMeasInfoCor	−2.27 × 10^−1^ (−2.44 × 10^−1^–−2.18 × 10^−1^)	−2.04 × 10^−1^ (−2.41 × 10^−1^–−1.86 × 10^−1^)	0.151	
SecMeasInfoCor	9.50 × 10^−1^ (9.45 × 10^−1^–9.62 × 10^−1^)	9.24 × 10^−1^ (9.11 × 10^−1^–9.54 × 10^−1^)	0.024	
InvDiffMom	1.69 × 10^−1^ (1.43 × 10^−1^–1.79 × 10^−1^)	2.31 × 10^−1^ (2.13 × 10^−1^–2.38 × 10^−1^)	0.000	
InvDiffMomNorm	9.91 × 10^−1^ (9.90 × 10^−1^–9.93 × 10^−1^)	9.96 × 10^−1^ (9.95 × 10^−1^–9.97 × 10^−1^)	0.000	
InvDiff	2.59 × 10^−1^ (2.32 × 10^−1^–2.68 × 10^−1^)	3.19 × 10^−1^ (3.02 × 10^−1^–3.25 × 10^−1^)	0.000	
InvDiffNorm	9.40 × 10^−1^ (9.35 × 10^−1^–9.48 × 10^−1^)	9.59 × 10^−1^ (9.55 × 10^−1^–9.62 × 10^−1^)	0.000	
InvVar	1.69 × 10^−1^ (1.49 × 10^−1^–1.80 × 10^−1^)	2.37 × 10^−1^ (2.19 × 10^−1^–2.49 × 10^−1^)	0.000	
JointMax	7.47 × 10^−3^ (6.04 × 10^−3^–9.02 × 10^−3^)	1.17 × 10^−2^ (1.03 × 10^−2^–1.45 × 10^−2^)	0.000	
SumAvg	8.80 × 10^1^ (8.20 × 10^1^–9.28 × 10^1^)	9.11 × 10^1^ (8.76 × 10^1^–9.35 × 10^1^)	0.256	
SumEnt	6.06 × 10^0^ (5.99 × 10^0^–6.23 × 10^0^)	5.65 × 10^0^ (5.52 × 10^0^–5.76 × 10^0^)	0.000	
JointVar	1.17 × 10^2^ (9.70 × 10^1^–1.50 × 10^2^)	5.86 × 10^1^ (4.86 × 10^1^–7.32 × 10^1^)	0.000	
ShortRunEmph	9.64 × 10^−1^ (9.63 × 10^−1^–9.71 × 10^−1^)	9.50 × 10^−1^ (9.48 × 10^−1^–9.54 × 10^−1^)	0.000	gray level run length matrix
LongRunEmph	1.16 × 10^0^ (1.14 × 10^0^–1.16 × 10^0^)	1.23 × 10^0^ (1.21 × 10^0^–1.24 × 10^0^)	0.000	
GLNU	4.40 × 10^1^ (3.80 × 10^1^–4.60 × 10^1^)	5.88 × 10^1^ (4.65 × 10^1^–6.83 × 10^1^)	0.004	
GLNUnorm	3.19 × 10^−2^ (2.96 × 10^−2^–3.43 × 10^−2^)	4.29 × 10^−2^ (3.97 × 10^−2^–4.60 × 10^−2^)	0.000	
RunLenNU	1.24 × 10^3^ (1.13 × 10^3^–1.30 × 10^3^)	1.26 × 10^3^ (1.09 × 10^3^–1.41 × 10^3^)	0.774	
RunLenNUnorm	9.09 × 10^−1^ (9.06 × 10^−1^–9.27 × 10^−1^)	8.77 × 10^−1^ (8.73 × 10^−1^–8.85 × 10^−1^)	0.000	
RunPerc	9.52 × 10^−1^ (9.50 × 10^−1^–9.59 × 10^−1^)	9.34 × 10^−1^ (9.30 × 10^−1^–9.38 × 10^−1^)	0.000	
GLVar	1.24 × 10^2^ (1.06 × 10^2^–1.49 × 10^2^)	6.35 × 10^1^ (5.31 × 10^1^–7.94 × 10^1^)	0.000	
RunLenVar	5.38 × 10^−2^ (4.86 × 10^−2^–5.64 × 10^−2^)	7.76 × 10^−2^ (7.16 × 10^−2^–8.55 × 10^−2^)	0.000	
RunEntr	5.55 × 10^0^ (5.45 × 10^0^–5.71 × 10^0^)	5.21 × 10^0^ (5.10 × 10^0^–5.33 × 10^0^)	0.000	
LowGLRunEmph	1.40 × 10^−3^ (6.75 × 10^−4^–2.20 × 10^−3^)	5.94 × 10^−4^ (5.31 × 10^−4^–7.09 × 10^−4^)	0.000	
HighGLRunEmph	2.00 × 10^3^ (1.75 × 10^3^–2.25 × 10^3^)	2.13 × 10^3^ (1.94 × 10^3^–2.25 × 10^3^)	0.503	
ShortRunLowGLEmph	1.38 × 10^−3^ (6.55 × 10^−4^–2.17 × 10^−3^)	5.69 × 10^−4^ (5.05 × 10^−4^–6.85 × 10^−4^)	0.000	
ShortRunHighGLEmph	1.93 × 10^3^ (1.70 × 10^3^–2.17 × 10^3^)	2.01 × 10^3^ (1.84 × 10^3^–2.11 × 10^3^)	0.753	
LongRunLowGLEmph	1.50 × 10^−3^ (7.66 × 10^−4^–2.32 × 10^−3^)	7.10 × 10^−4^ (6.53 × 10^−4^–8.17 × 10^−4^)	0.002	
LongRunHighGLEmph	2.31 × 10^3^ (2.09 × 10^3^–2.61 × 10^3^)	2.64 × 10^3^ (2.44 × 10^3^–2.80 × 10^3^)	0.065	

Note: Mean values with standard deviation (SD) are reported for normally distributed variables; otherwise, median values with interquartile range (IQR) are reported. Healthy individuals (*n* = 10) in Cluster 2 were missing some clinical measurements (SBP, DBP, eGFR slope, urine protein, and blood glucose) and were excluded from the respective statistical analysis. Abbreviations: CKD: chronic kidney disease; CKD-EPI: the CKD Epidemiology Collaboration; eGFR: estimated glomerular filtration rate; SBP: systolic blood pressure; DBP: diastolic blood pressure; BMI: body mass index.

**Table 4:** Logistic regression models to predict rapid vs. non-rapid progressors in CKD.

Model Features	Features	Sensitivity	Specificity	AUC-ROC
Radiomics	GLCMContr, SumEnt, CoV, FirstMeasInfoCor	71%	43%	0.75
Clinical	24 h urine protein excretion, sex	57%	91%	0.94
Combination *	24 h urine protein excretion, sex, AuCor	57%	96%	0.96

Note: For individuals missing protein excretion measurements (*n* = 4), the value was imputed based upon the mode of the data (0 g/day). * indicates a combination of radiomic and baseline clinical features. AUC-ROC: area under the curve of the receiver operating curve.

## Data Availability

The data presented in this study may be available on request from the corresponding author. The data are not publicly available due to lack of prior consent by the participants.
